# Wireless Sensor Node Self-Powered by a Hybrid-Supercapacitor and a Multi-Junction Solar Module

**DOI:** 10.3390/s26051475

**Published:** 2026-02-26

**Authors:** Mara Bruzzi, Irene Cappelli, Mirko Brianzi, Carlo Cialdai, Ada Fort, Valerio Vignoli

**Affiliations:** 1 Department of Physics and Astronomy, University of Firenze, 50019 Sesto Fiorentino, Italy; 2 Istituto Nazionale di Astrofisica (INFN), Sezione di Firenze, 50019 Sesto Fiorentino, Italy; mirko.branzi@fi.infn.it (M.B.); carlo.cialdai@fi.infn.it (C.C.); 3 Department of Information Engineering and Mathematics, University of Siena, 53100 Siena, Italy; cappelli@diism.unisi.it (I.C.); ada.fort@unisi.it (A.F.); valerio.vignoli@unisi.it (V.V.)

**Keywords:** wireless sensor nodes, CO_2_ sensors, multi-junction photovoltaic, hybrid supercapacitors, self-powered sensors, environmental gas monitoring, IoT, photosynthesis monitoring

## Abstract

This work presents a compact, self-powered wireless CO_2_ sensing node for autonomous environmental monitoring. The system integrates a high-efficiency multijunction photovoltaic (PV) module, a 4000 F hybrid supercapacitor operating at 3.6–4.2 V, and a custom power management system in a LiPo-sized form factor. The PV module, composed of nine parallel triple-junction solar cells, achieves an average efficiency of 27% and delivers peak power at 4.26 V under 600 W/m^2^ irradiance. The sensing unit includes miniaturized CO_2_, humidity, and temperature sensors with LoRa-based wireless communication. The low-power NDIR CO_2_ sensor provides a resolution of 15–20 ppm and a response time of ~45 s. Week-long tests demonstrated fully autonomous operation with reliable 5 min data transmission, capturing diurnal CO_2_ variations associated with plant activity even under low irradiance. Energy storage occurs for irradiance levels ≥65 W/m^2^, and long-term simulations confirm stable supercapacitor voltage over yearly cycles. This work demonstrates a compact multijunction solar–hybrid supercapacitor platform capable of sustaining WSN for long-term, maintenance-free CO_2_ monitoring under real-world and low-irradiance conditions. Our results demonstrate that the sensing node can reliably monitor plant-driven CO_2_ dynamics, clearly resolving the expected photosynthesis–respiration cycles and their dependence on incident solar radiation, while simultaneously sustaining its energy budget under highly challenging illumination and transmission conditions.

## 1. Introduction

Air quality monitoring has increasingly relied on the deployment of extended networks of wireless sensor nodes (WSNs) composed of miniaturized and energy-sustainable devices [1,2,3,4,5,6,7]. In these systems, each node typically integrates an energy harvesting and storage section to support long-term autonomous operation [7,8,9]. The overall energy budget of a WSN node is primarily determined by the sensing modality and by wireless data transmission tasks. Among the monitored air-quality parameters, carbon dioxide (CO_2_) has emerged as a key indicator, owing to its relevance for indoor air quality assessment, ventilation control, environmental monitoring, and the analysis of biological and anthropogenic processes. As a consequence, CO_2_ monitoring currently represents one of the most widespread and demanding sensing functions within air-quality-oriented WSNs.

From an energy perspective, CO_2_ sensors are generally characterized by relatively high power consumption [10], which makes their integration into ultra-low-power WSNs particularly challenging. Most high-accuracy CO_2_ sensors are based on Non-Dispersive Infrared (NDIR) technology, which relies on an infrared source that was traditionally implemented using incandescent lamps, typically requiring power levels up to approximately 300 mW [11]. Alternative sensing technologies, such as chemiresistive sensors, also exhibit comparable power consumption due to their high-temperature operation [12], while particulate matter sensors may require power levels that are even one order of magnitude higher [13]. More recently, solid-state infrared LED sources operated in pulsed mode have become available, enabling a significant reduction in average power consumption to the milliwatt range and opening new opportunities for the deployment of CO_2_ sensing nodes in energy-constrained WSNs [14].

Despite the extensive literature on energy-harvesting wireless sensor nodes for environmental monitoring, achieving long-term energy autonomy remains challenging, especially when gas sensing is involved. Most existing WSN solutions rely on conventional silicon-based photovoltaic modules, characterized by a 10–20% efficiency depending on their crystalline quality [15], requiring a non-negligible size, often coupled with electrochemical batteries [1,2,3,4,5,6,7,8,9].

While effective in moderate illumination conditions, these approaches often result in relatively large harvesting areas, limited lifetime due to battery aging, and the need for periodic maintenance, which is incompatible with dense, long-term, or hard-to-access deployments. Due to the power requirements, the problem becomes more critical for CO_2_ monitoring [10,11]. Even recent low-power NDIR devices based on pulsed LED sources, although reducing average power consumption to the milliwatt range, still require the power supply to sustain short but intense current peaks and to guarantee stable operation over extended periods [12,13]. As a consequence, the integration of CO_2_ sensing in self-powered WSNs is often limited or requires operating conditions and energy budgets that restrict long-term autonomy. Several works have explored alternative harvesting strategies or storage technologies, such as supercapacitors or hybrid storage elements, to overcome battery-related limitations [16,17,18,19,20]. However, their integration with CO_2_ sensing nodes operating under low or highly variable irradiance—such as diffuse light, indoor–outdoor transitions, or winter conditions—has been only partially addressed, and experimental demonstrations under realistic long-term conditions remain scarce.

In this context, the present work addresses the need for a compact and maintenance-free wireless CO_2_ sensing node capable of sustained autonomous operation under unfavorable illumination conditions. The proposed solution combines a high-efficiency GaAs-based multi-junction (MJ) photovoltaic (PV) module, achieving almost 30% efficiency under terrestrial solar irradiation, which significantly reduces the required harvesting area compared to silicon-based devices [16,21], with a hybrid supercapacitor (SC) providing high power density, virtually unlimited cycling capability, and robustness against the pulsed current demands of NDIR sensing. The system-level integration of these elements with a duty-cycled LoRa-based communication architecture is experimentally validated through laboratory tests and extended deployments, including winter operation, demonstrating continuous autonomous functionality and reliable CO_2_ monitoring.

The developed compact WSN hosts a solid-state LED-based sensing device for CO_2_ monitoring [14], plus temperature and relative humidity sensors. The energy harvesting and storage system is based on a hybrid supercapacitor, characterized by high capacitance, power and energy densities, low discharge current and exceptionally high cycling (10^4^) [16,17,18,19,20], handled by a high-current (1 A) miniaturized Power Management System (PMS). The entire system, with a total size of about 20 cm^2^, has been demonstrated to be self-powered for sustained, virtually unlimited outdoor performance. The primary objective of this activity was to assess the feasibility of long-term, autonomous operation of a low-cost CO_2_ sensing node under continuous sampling conditions. The agricultural use case, and particularly the monitoring of plant-related CO_2_ dynamics, is therefore adopted as a representative and meaningful application scenario to stress the system and demonstrate its ability to operate reliably over extended periods while capturing plant-driven CO_2_ variations.

System architecture and single elements characterization are presented in Section 2 (Materials and Methods). Section 3 describes the main experimental results obtained with the whole prototype in laboratory, both with a Sun Simulator lamp and under solar radiation. From these results, the same section also shows the extrapolated WSN power autonomy during virtually unlimited operation. Finally, the conclusions and outlooks of the presented research are reported in Section 4.

## 2. Materials and Methods

**Figure 1:**
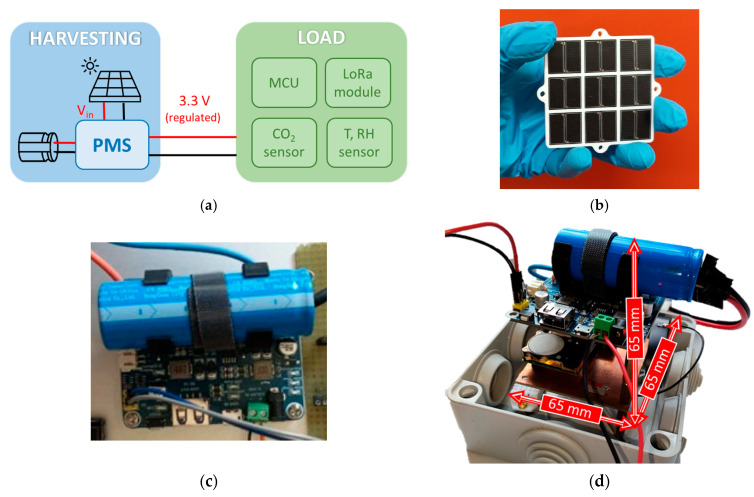
(**a**) Schematic block diagram of the designed sensor node; (**b**) photograph of the 3 × 3 PV module composed of 18 triple-junction solar cells; (**c**) photograph of the hybrid supercapacitor and PMS; (**d**) photograph of the entire system with dimensions.

A sketch of the device architecture and pictures of the realized system are shown in Figure 1a–d. The system is composed of the following:

1. An energy harvesting-storage section comprising a PV module, a hybrid supercapacitor and a power manager board;
2. A load section, carrying the sensors and the transmission device.

In the following sections we describe each of the elements composing the entire device.

### 2.1. Energy Harvesting-Storage Section

#### 2.1.1. PV Module

The PV module used in this work is composed of nine solar cells produced by Shanghai YIM Machinery Equipment Co., Ltd. (MSCM-4.5-14.8-30%, Shanghai, China) [22]. Each solar cell is a series of two triple junctions grown by the GaAs wafer technology. They are dust and waterproof as from IP67 protocol. A set of relevant parameters are listed in Table 1 under terrestrial applications and T = 25 °C.

We have prototyped a PV module in 3 × 3 configuration for a total of 18 triple junctions with an active area of 18.6 cm^2^. All the 9 solar cells have been connected in parallel, to maximize the output current and to get a peak voltage close to the max operation voltage of the battery (4.2 V). A picture of 3 × 3 PV module is shown in Figure 1b. The total active area is 25.1 cm^2^. The module conversion efficiency has been tested outdoors and in laboratory under a Sun Simulator (Abet Technology 2000 Milford, CT, USA) based on a 150 W Xe lamp [23]. A Keithley 2401 source/electrometer (Cleveland, OH, USA) has been used to read out the current–voltage characteristics of the PV module. The instrument is characterized by a maximum measured voltage V_max_ = 21 V, a 100 μV resolution, an accuracy ΔV = 0.015% of reading +1.5 mV and maximum current supplied I = 105 mA. Radiation intensity and module temperature have been monitored by a Kipp & Zonen CMP3 pyranometer (Delft, The Netherlands) equipped with a SOLRAD readout system [24] and a Pt1000 thermometer. The pyranometer sensitivity is S = 28 ± 4 μV/(W/m^2^), its spectral range is 300–2800 nm, and the typical response time is 1 s.

**Figure 2:**
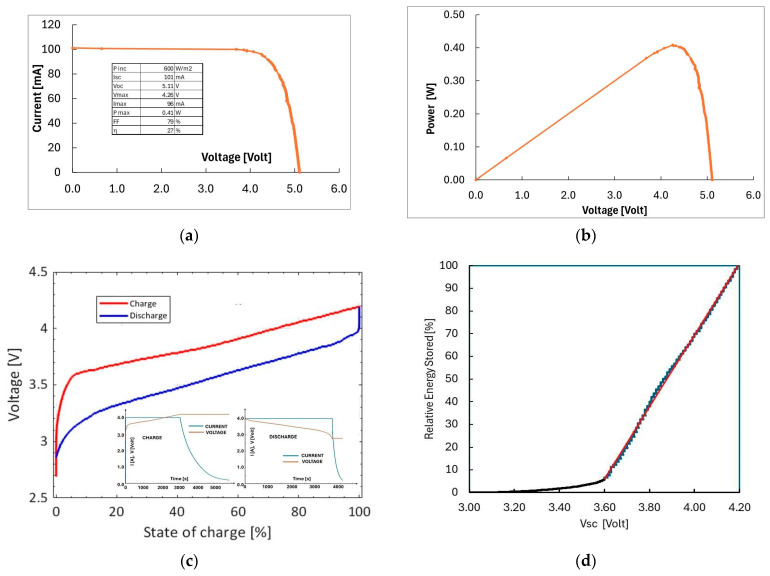
(**a**) I–V characteristics of the 3 × 3 PV module measured outdoors in clear-sky conditions, 600 W/m^2^, 37° tilt T = 30 °C. (**b**) P–V characteristics of the 3 × 3 PV module as calculated from the data of (**a**). (**c**) Charge-discharge curves of the hybrid supercapacitor. Inset: time diagrams for charge-discharge current and voltage. (**d**) Relative energy stored in the supercapacitor as a function of the voltage across the electrodes, V_SC_, in the operation range 3.0–4.2 V. Best fit of the stored energy in the range 3.6–4.2 V is shown as a red line.

As an example, the current–voltage (I–V) characteristics under 600 W/m^2^ intensity measured with the PV module outdoors, on a clear-sky day in winter (February 2025) in Florence, Italy, is shown in Figure 2a. The temperature during the measurement was 30 °C ± 0.9 °C. The corresponding power–voltage (P–V) curve is shown in Figure 2b. Relevant photovoltaic parameters are reported in the inset of Figure 2a. The maximum power at this intensity at about 0.4 W and corresponding to an efficiency of 27% is obtained at V_max_ = 4.26 V.

#### 2.1.2. Hybrid Supercapacitor and PMS

As a storage unit we used a hybrid supercapacitor manufactured by DongGuan GongHe Electronics Co., Ltd. (C424000R, Dongguan, China) with a cylindrical shape, 69 mm in height and 24 mm in diameter. It is characterized by a protection class IP30 and a weight of 70 g [25]. The nominal capacitance is 4000 F, the maximum voltage is 4.2 V while the energy storage is 14 Wh. It is characterized by a low discharge current ≤0.5 mA/72 h, a cycle lifetime of 10^4^ and an operating temperature range −40–65 °C. The measured charge-discharge behavior is shown in Figure 2c; it has been obtained using the current and voltage profiles in time shown in the inset. The internal resistance of the device is calculated as about 20 mΩ by the difference between the charging-discharging curve [16]. The energy stored in the supercapacitor, obtained as $U=\frac{1}{2}QV_{SC}$ (where V_SC_ is the voltage across the supercapacitor) represented as a function of V_SC_, looks almost linear in the range 3.7–4.2 V, as evidenced in Figure 2d. Best fit of the stored energy as a function of supercapacitor voltage V_SC_ in this linear range is $U(V_{SC})$ = 55.936 $V_{SC}$ −199.25 J as given in [16] for the same hybrid supercapacitor.

The PMS used in our device is from Waveshare, Shenzen, China [26] based on the CN3791 solar power management chip for solar panel charging and buck input. It has a 65.2 mm × 56.2 mm size, the voltage input from PV module can be selected from 6 V to 24 V, and the operation temperature range is −40–80 °C. It is designed to support a 14,500 Li-ion battery (850 mAh) but we customized its electrodes to fit the C424000R hybrid supercapacitor, as shown in Figure 1c. The voltage across the storage element must be within 2.9 V and 4.2 V. The load section of the device is connected to its 3.3 V/1 A regulated output. The Maximum Power Point (MPP) is automatically selected from V_in_ as a fraction of the open circuit voltage V_OC_ by a voltage divider. As explained in the CN3791 datasheet, the PMS regulates the MPP voltage through an off-chip resistor divider, by means of the following equation: V_MPPT_ = V_MAX_ − V_OC_ = 1.205 × (1 + R_3_/R_4_) with R_3_ = 10 kΩ and R_4_ depending on the selected V_OC_ (6 to 24 V). In our case, selecting V_OC_ = 6 V, the actual voltage output fixed by the PMS is V_OUT_ = 4.28 V, very close to the actual maximum power voltage of the module and similar to the maximum operating voltage of the battery (4.2 V).

### 2.2. Load Section

The sensor node we developed is a low-power platform designed for continuous environmental monitoring, integrating multiple sensing elements with long-range wireless communication. The final goal is to enable continuous data acquisition in distributed or hard-to-reach locations. Environmental monitoring increasingly requires sensors capable of capturing high-resolution spatiotemporal variations in key atmospheric parameters, and the proposed node addresses this need by integrating temperature, humidity, and CO_2_ measurements within a single low-power device. Such parameters are fundamental indicators of microclimatic conditions, indoor air quality, and ecosystem dynamics, and their joint observation provides a more comprehensive picture of the environmental status than single-sensor solutions.

At its core, the system employs an ATmega-based microcontroller (by Microchip Technology, Chandler, AZ, USA), chosen for its low energy consumption, stable performance in embedded applications, and compatibility with a wide range of peripheral interfaces. The microcontroller is responsible for coordinating the sensing tasks, acquiring and preprocessing data, and managing the timing of wireless transmissions.

Environmental parameters are measured using two types of sensors. Temperature and relative humidity are obtained from a DHT22 digital sensor (Adafruit, New York, NY, USA), which provides factory-calibrated output with 16-bit resolution and typical accuracies of ±0.5 °C and ±2% RH, respectively. Its single-wire communication protocol minimizes I/O requirements and simplifies integration with the microcontroller. Carbon dioxide concentration is monitored using a CozIR-LP NDIR sensor (GSS, Cumbernauld, UK) [14], specifically designed for low-power applications. For the application considered, the sensor was programmed to acquire one sample every 500 ms and to average multiple readings. More details about this sensor are reported in the following subsections.

For wireless connectivity, the node uses a LoRa (Long Range) radio transceiver operating in the sub-GHz ISM band (e.g., RFM95 LoRa module by HopeRF, Shenzhen, China). LoRa modulation provides a high link budget and strong robustness against interference, enabling reliable transmission over several kilometers depending on the deployment environment. Its configurable spreading factor (SF) allows balancing data rate and communication range, while inherently low duty cycle contributes to extending the node energy autonomy. During the performed tests, transmitting frequency 868 MHz, output power 14 dBm, coding rate (CR) 4/5, SF 7, and bandwidth (BW) 125 kHz were set.

The operational cycle consists of periodic wakeups of the microcontroller, during which the sensors are powered, measurements are acquired, and a formatted data packet is transmitted through the LoRa interface. After completing these tasks, the system enters a deep-sleep state to minimize energy consumption. This duty-cycled operation enables long-term deployment in remote locations where battery replacement or maintenance is limited. During the conducted tests, the node remained active for about 40 s, while the sleep period (i.e., the time window between two system activations and radio transmissions) was dynamically set by the user.

The average current consumption of the system as a whole was measured in both active and sleep phases to quantify its overall energy demand. During the active phase, which includes sensor acquisition and the LoRa radio transmission, the latter being the most energy-intensive operation with current peaks up to approximately 130 mA, the node exhibits an average current draw of 7.35 mA. Given that the active period lasts about 40 s, the corresponding charge consumption is approximately 300 mC, while the associated energy consumption, assuming a supply voltage of 3.3 V, amounts to roughly 970 mJ. When the system enters sleep mode, the current drops significantly to 247 µA at 3.3 V. For a representative sleep interval of 5 min (300 s), this corresponds to a charge consumption of about 74 mC.

#### 2.2.1. CO_2_ Sensor: Description

**Figure 3:**
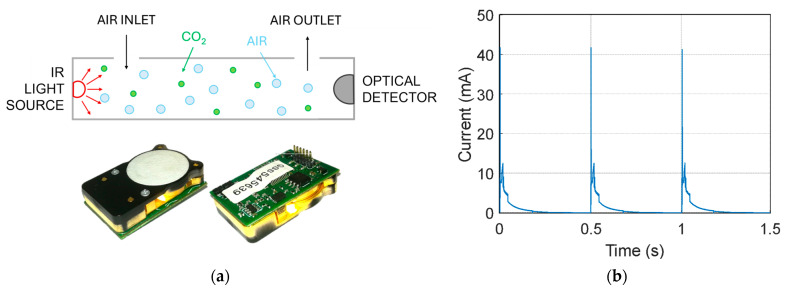
(**a**) Typical working principle of an NDIR sensor and photograph of the CozIR-LP sensor. (**b**) Pulse current profile during sensor measurement cycles acquired using a Nordic Power Profiler Kit II operated as a source meter at 3.3 V with a sampling frequency of 100 kHz.

The CO_2_ sensor used in this work is the CozIR-LP from Gas Sensing Solutions [14]. It is a low-power NDIR device that employs LED-based optical technology operated in pulsed mode, significantly reducing energy consumption and warm-up time compared to traditional filament-lamp NDIR sensors [27]. The typical working principle of an NDIR CO_2_ sensor and a picture of the CozIR-LP are shown in Figure 3a. The device supports measurement ranges up to 5000 ppm, suitable for applications of air quality or environmental monitoring. Communication with the microcontroller is performed via a UART interface, ensuring robust digital data transfer and allowing access to additional functionalities such as zero calibration, filter setting and configurable sampling rates. Moreover, the device supports multiple energy-saving modes that allow periodic sampling, while maintaining reduced current consumption, and a sleep state where the sensor remains powered without performing readings. The internal digital filtering stage stabilizes the CO_2_ measurements by attenuating high-frequency noise present in the raw optical signal and provides more reliable readings. The filtering level can be adjusted via the UART interface, allowing a trade-off between responsiveness and noise suppression: lower filtering yields faster reaction to concentration changes, whereas higher filtering provides a more stable but slower output.

Real-time sensing capability, very low heat dissipation, small size (approximately 3 cm side) and weight, as well as its reduced cost, make the CozIR-LP particularly suitable for battery-powered WSN nodes. Its low quiescent current, typically in the order of tens of microamperes in idle mode, enables long-term deployment under duty-cycled operation. Table 2 shows the main parameters of the sensor [14].

The CozIR-LP exhibits a characteristic pattern of current consumption over each measurement cycle. The sensor uses a pulsed-LED NDIR optical architecture: during each measurement, the internal LED is briefly driven, producing peak current spikes that are substantially higher than the quiescent or idle current during normal operation or in between readings. The presence of these current peaks affects overall energy budgeting, and the power management sub-system must sustain them: although each measurement only lasts a short time (about 200 ms) and the duty cycle of LED activation is low (about 20%), the instantaneous current peak must be accounted for in the design of the power supply (batteries or supercapacitors). By tuning the acquisition interval, one can optimize the trade-off between measurement noise reduction and energy efficiency, a critical aspect for wireless sensor nodes where power budget is a limiting factor. The current profile of the CO_2_ sensor alone during the measurement cycle is reported in Figure 3b. The current was acquired using a Nordic Semiconductor (Trondheim, Norway) Power Profiler Kit II operated as a source meter at 3.3 V, with a sampling frequency of 100 kHz, in order to capture the fast transient peaks associated with the NDIR emitter activation. As specified in the CozIR-LP datasheet [14], the current profile includes short turn-on peaks of up to about 40 mA and sampling-related peaks around 15 mA, while the average current remains on the order of 1 mA. Therefore, the peak amplitudes, pulse structure, and average current observed in Figure 3b are fully consistent with these specifications, confirming that the measured profile reflects the expected sensor operation.

#### 2.2.2. CO_2_ Sensor: Characterization

**Figure 4:**
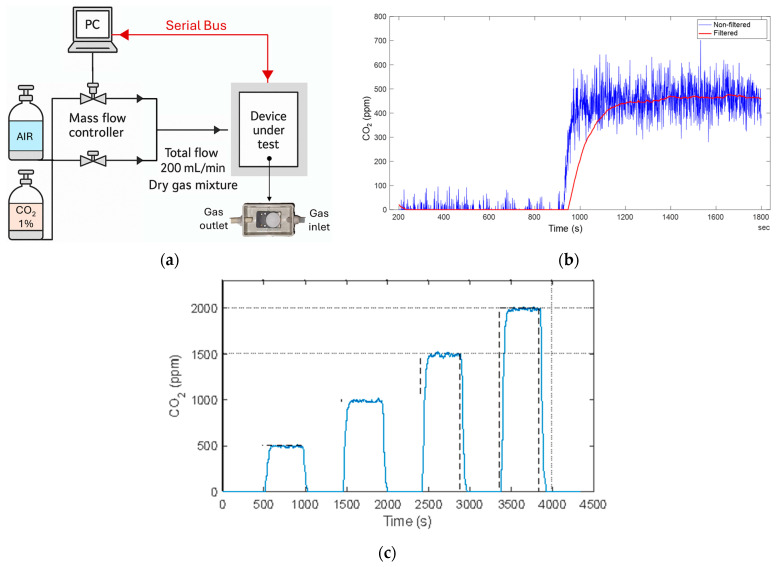
(**a**) Characterization set-up of the CO_2_ sensor; (**b**) unfiltered and filtered (filter parameter *n* set to 128) concentration signals for a step change from 0 ppm to 500 ppm (as per legend); (**c**) sensor response during a step-response test in which the CO_2_ concentration is repeatedly increased from 0 ppm to 500–1000–1500–2000 ppm, and back to 0 ppm each time.

Prior to assembling in the WSN system, the CO_2_ sensor underwent a characterization procedure under known concentrations of CO_2_ to calibrate it and to determine its performance in terms of sensitivity and response time. A dedicated gas mixing setup, schematically reported in Figure 4a, was used to generate well-controlled CO_2_ concentrations. The calibration system consisted of mass flow controllers operated via a PC, which allowed independent adjustment of the total flow and of the gas composition.

The flow controllers were connected to certified cylinders of dry synthetic air and CO_2_ (1% nominal concentration). By varying the relative flow rates while keeping the overall flow fixed at 200 mL/min, it was possible to produce a sequence of dry gas mixtures with precisely defined CO_2_ levels. This approach ensured a stable and reproducible environment for characterizing the sensor response. Throughout tests, the sensor output was continuously recorded via the serial interface. To this aim, a custom LabVIEW (version 2016) routine supervised both the acquisition and the actuation of the flow controllers, guaranteeing that changes in gas composition and data logging were synchronized. This integrated setup enabled accurate mapping of the sensor behavior across the chosen concentration range, providing the reference values required for subsequent validation.

Moreover, the CO_2_ sensor embeds an internal low-pass filter with a user-configurable setting parameter n. Although the exact internal realization of the low-pass filter is not disclosed by the manufacturer, its behavior can be reasonably interpreted as equivalent to a moving-average filter operating on a finite number of samples n. Under this assumption, white measurement noise is attenuated with a power reduction proportional to the inverse of the number of averaged samples n, while the standard deviation of the measured signal and therefore the effective measurement resolution scales as $1/\sqrt{n}$. Conversely, the effective time constant of the filtered response increases linearly with n. This is confirmed by the data reported in Figure 4b, which shows both the unfiltered and the filtered (filter parameter *n* set to 128) concentration signals for a step change from 0 ppm to 500 ppm. Filtering effectively reduces noise and improves the signal-to-noise ratio of the response, but it also leads to a marked increase in rise-time. However, it is worth noting that part of the observed rise-time increase is not solely due to digital filtering. Indeed, the non-filtered signal already exhibits a relatively slow response, indicating intrinsic dynamics due to gas diffusion and air mixing in the measurement chamber.

The noise floor and the sensor resolution can be estimated from the unfiltered signal under nominally constant flow conditions reported in Figure 4b. In detail, short-term fluctuations due to high-frequency noise are recorded, within a peak-to-peak range of about 200 ppm, thus yielding a corresponding noise of approximately 30–35 ppm rms. The filter setting should therefore provide an appropriate balance between noise suppression and responsiveness, improving long-term stability without compromising the interpretability of the measured concentration trends. Moreover, the filter setting for experimental deployment must be chosen considering a minimum acceptable response time dictated by the application requirements.

In particular, the parameter n= 32 was selected as a good compromise between noise reduction and response time and this setting was subsequently used in the following tests. Given that the CO_2_ sensor produces one raw measurement every 500 ms, the selected filter setting of 32 can be interpreted as averaging 32 consecutive samples, yielding an effective integration time of approximately 16 s. This results in an improvement of measurement resolution by a factor $\sqrt{32}\;\approx 5.7$, at the expense of a corresponding reduction in temporal resolution. The residual noise is thus reduced to approximately 5–6 ppm rms with respect to the unfiltered case, yielding an effective resolution in the order of 15–20 ppm. This trade-off is acceptable for the targeted application, given that the phenomena of interest in this work, i.e., diurnal photosynthesis–respiration cycles, evolve on time scales of several minutes or longer.

Concerning rise-time estimation, the step-response test reported in Figure 4c can help in providing a qualitative assessment of the system response time when used with a controlled flow. In detail, the sensor was tested with varying concentration steps alternating increasing gas concentrations (i.e., 500 ppm, 1000 ppm, 1500 ppm, 2000 ppm) to zero target gas phases. The results demonstrate that the sensor maintains stable baseline conditions, with no noticeable residual offset or significant drift, and it exhibits repeatable responses to repeated exposures, with a smooth and monotonic transition toward the new steady-state value, without overshoot or oscillations. Moreover, the measured average $T_{10-90}$ transition time is approximately 45 s, which is fully compatible with the target application, where CO_2_ dynamics evolve on much slower time scales, confirming that the proposed system can reliably track concentration changes relevant to environmental and plant-related monitoring.

As the sensing element is a commercially available device, its accuracy specifications are primarily defined by the manufacturer’s datasheet, which reports an accuracy on the order of ±(30 ppm +3% of reading) over the 0–50 °C temperature range after zero calibration at room temperature [14]. Within this framework, additional laboratory tests were performed to verify the consistency of the sensor behavior with the declared specifications. A multi-point calibration test was performed, where the sensor was exposed to a set of known gas concentrations covering the full operating range, from 0 ppm to 5000 ppm at nominal steps of 500 ppm. After calibration, a root-mean-square error of about 40 ppm and a mean absolute error of about 35 ppm were found, while the absolute error remained within 50 ppm even at the highest concentration levels, confirming the data from the manufacturer and the datasheet specifications. Moreover, the results proved that the sensor response is linear over the full operating range, justifying the use of a first-order calibration model in addition to the factory calibration foreseen for the zero-parameter setting only. Indeed, it is worth noting that the employed CO_2_ sensor provides an internal correction mechanism only for zero setting, while no internal span calibration is implemented. For this reason, further calibration, apart from the factory one, was performed with a two-points calibration procedure by exposing the device to gas mixtures to two known concentrations: 0 ppm to set the zero parameter and 2000 ppm to fix the sensor span. During this procedure sufficient time was allowed between the two gas phases to allow the sensor signal to reach a stable response. From a metrological perspective, this calibration can effectively compensate for shifts in offset or gain due to aging, installation-dependent effects, and environmental variations.

Once this laboratory calibration was performed, preliminary long-term measurements were carried out both outdoors and in an indoor environment to evaluate the behavior of the sensor under real operating conditions. During these tests, the sensor was used standalone, without the entire monitoring system, and its readings were directly acquired via serial interface by using a LabVIEW VI (version 2016). Within this system-level framework, stability is therefore addressed from an application-oriented perspective, focusing on long-term and operational robustness under the performed measurement campaigns. Moreover, the reliability of the measurements is further supported by the initial laboratory testing performed using reference concentrations from the mass flow controller bench and the certified gas cylinders.

**Figure 5:**
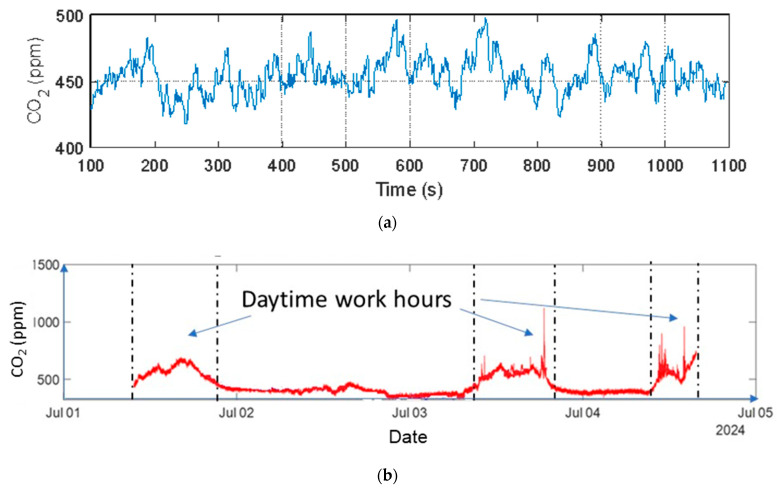
(**a**) Outdoor and (**b**) indoor CO_2_ measurements as a function of time acquired during preliminary tests.

The measurements collected are reported in Figure 5. Outdoor tests confirmed that the sensor remains stable in open-air conditions, where CO_2_ levels exhibit only slow natural fluctuations also ascribable to the effect of the airflow. Indoor measurements were then performed to verify the sensor ability to detect variations associated with human presence. In this case, the sensor clearly captured the CO_2_ increase during occupancy periods and the gradual decrease during the weekend when the room was unoccupied. These preliminary results proved that the system is sufficiently stable outdoors and sensitive enough to track occupancy-related CO_2_ dynamics indoors. Moreover, the CO_2_ measurements did not exhibit noticeable baseline drift, instability, or degradation of performance. Based on these encouraging results, extended tests were carried out using the complete sensing node, including both the monitoring and energy-harvesting subsystems. Additional details about these experiments are provided in Section 3.

## 3. Results

The performances of the complete prototype, including both energy-harvesting section and load, as described above, have been studied both in laboratory and outdoor settings. The energy accumulated in the supercapacitor depends linearly on V_SC_ in the range 3.7–4.2 V (see Figure 2d). So, to determine energy accumulation or dissipation in the battery during operation, it is sufficient to measure in real time the voltage across the supercapacitor, V_SC_, in this range.

### 3.1. Prototype Characterization in Laboratory

**Figure 6:**
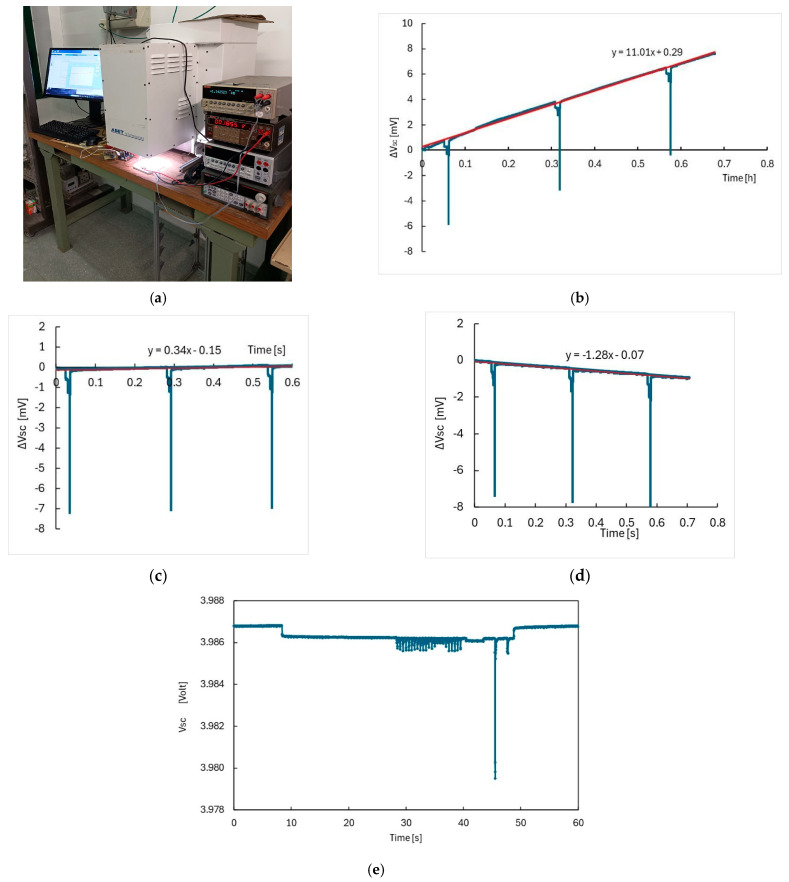
(**a**) Picture of the setup for laboratory tests. (**b**) V_SC_ variation (ΔV_SC_) vs. time measured during operation of the WSN under 500 W/m^2^ Sun Simulator intensity and Δt = 15 min transmission windows. Linear best fit to data used to determine the slope (dV_SC_)/dt is also shown. (**c**) Same as (**b**) in case of 100 W/m^2^ intensity; (**d**) Same as (**b**) in case of dark; (**e**) V_SC_ measured as a function of time during a sensing/transmitting phase.

When testing the complete sensor node in the laboratory, we placed the PV module under the Sun Simulator, at a fixed intensity and AM1.5G filtering conditions, and we measured the V_SC_ as a function of time for different transmission windows (see picture in Figure 6a). Due to the linear dependence of the stored energy on V_SC_, a decrease/increase of V_SC_ during operation will reveal discharging/charging of the battery.

In Figure 6b–d, we show the plot of the change in V_SC_, ΔV_SC_ = V_SC_(t) − V_SC_(0), versus time for transmission every Δt = 15 min under different illumination intensities. The linear best fit used to determine the slope during the time interval of three transmission phases is also shown. In the case of Figure 6b,c (500 W/m^2^ and 100 W/m^2^ intensity respectively), the positive slope of the linear best fit indicates a positive energy balance. In the case of dark, as shown in Figure 6d, we have $\frac{dV_{SC}}{dt}<0$, indicating that dissipation in the node is prevailing. These measurements have been repeated for different transmission time windows and different intensities, I, in order to get V_SC_(I, Δt) functions that can be used to parameterize the charge/discharge of the battery during variable intensity and in different transmission conditions.

Finally, Figure 6e shows V_SC_ as a function of time during a sensing/transmitting phase, lasting 40 s. At start/stop a step is observed due to switching off/on the PV module: this is caused by the internal resistance of the hybrid supercapacitor (about 20 mΩ, see [16]). During the sensing/transmitting phase, we recognize a set of pulses related to the CO_2_ sensing, followed by flat absorptions due to temperature/humidity measurement plus a steep peak caused by radio transmission. From data shown in Figure 6e, considering the linear relationship between the energy stored in the hybrid supercapacitor and V_SC_, we can evaluate the energy lost during each transmission: about 150 mJ in a time-frame of 20 s, corresponding to ~7 mW dissipated power.

### 3.2. Prototype Characterization Outdoors

**Figure 7:**
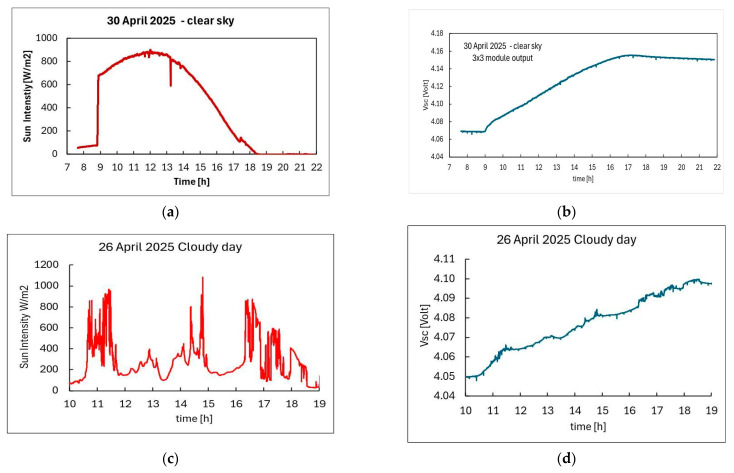
Measurement of V_SC_ as a function of time outdoors carried out in Florence, Italy during two different days in April 2025. (**a**) Sun radiation intensity measured on a clear-sky day (30 April 2025) vs. hour of the day; (**b**) V_SC_ measured during the same day in case of transmission every 15 min; (**c**) Sun radiation intensity measured on a cloudy day (26 April 2025) vs. hour of the day; (**d**) V_SC_ measured during the same day in case of transmission every 15 min.

A set of tests has been performed outdoors, in Florence, Italy. We placed the PV module directed to South, 37° tilted and we measured V_SC_ as a function of time for different transmission windows during the whole day. Meanwhile, Sun radiation intensity and temperature have also been monitored. Figure 7a,c show the Sun radiation intensity measured by the pyranometer in two days, one of clear sky (30 April 2025) and one with clouds (26 April 2025). Corresponding V_SC_(t) plots in case of sensing/transmission every 15 min are shown in Figure 7b,d respectively. In Figure 7b we observe an increase of V_SC_ when Sun light intensity is higher than approximately 100 W/m^2^, as indicated by measurements carried out indoors. In Figure 7d the slope of the V_SC_ vs. time curve is irregular, following the sudden changes of intensity due to cloud coverage during the day. Nonetheless, in both cases we have a significant increase of the stored energy at the end of the day.

### 3.3. Performance Evaluation Under Realistic Operating Conditions

To evaluate both the sensing performance and the energy autonomy of the proposed node also in worst-case conditions, a series of preliminary tests was conducted under controlled but realistic operating conditions. The experiments were carried out in the laboratory, with the PV module placed outside the window. In this configuration, the module receives direct sunlight only for a short period of the day and it must rely mostly on diffuse or low-angle light with respect to full-field outdoor deployment. Moreover, the tests have been conducted between November and December 2025, which represent the most unfavorable months of the year in terms of solar elevation, irradiance, and overall meteorological conditions. These factors significantly reduce the available solar energy, making the achievement of energy autonomy a particularly demanding task. In addition, the sensing node has been programmed with a relatively frequent transmission interval of five minutes, to evaluate its capability to operate autonomously despite a communication schedule that leads to non-negligible energy consumption. Such a configuration constitutes a stringent test scenario, as the radio subsystem is one of the major contributors to the overall power budget.

The sensing node was positioned inside a plexiglass enclosure containing a plant and illuminated with diffuse natural light, to monitor the CO_2_ dynamics associated with the plant activity. The enclosure was not intended to be airtight but rather to provide a semi-confined environment suitable for monitoring relative CO_2_ dynamics over time, while allowing for a limited gas exchange with the surrounding air. An additional CO_2_ sensor node was placed outside the chamber and used as an environmental reference for the monitoring of CO_2_ concentration in the laboratory. This configuration allows for potential air exchange between the enclosure and the surrounding room, since ambient-driven CO_2_ fluctuations would affect both the external and internal measurements. The comparison between the two nodes therefore provided a means to distinguish plant-induced CO_2_ dynamics from variations originating in the laboratory environment.

**Figure 8:**
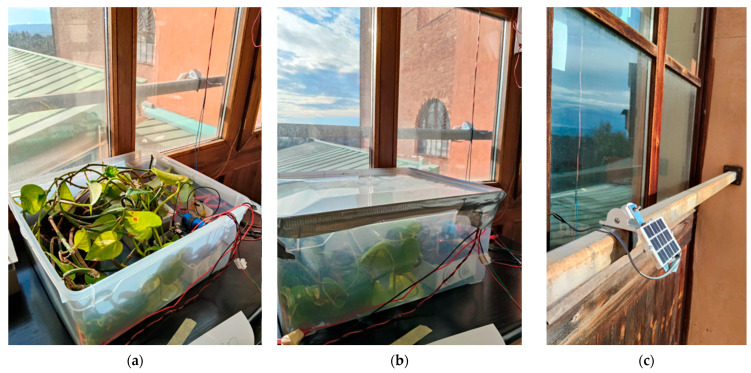
Setup used during the measurement campaign with plants: (**a**) plastic box open, (**b**) close, and (**c**) PV module.

**Figure 9:**
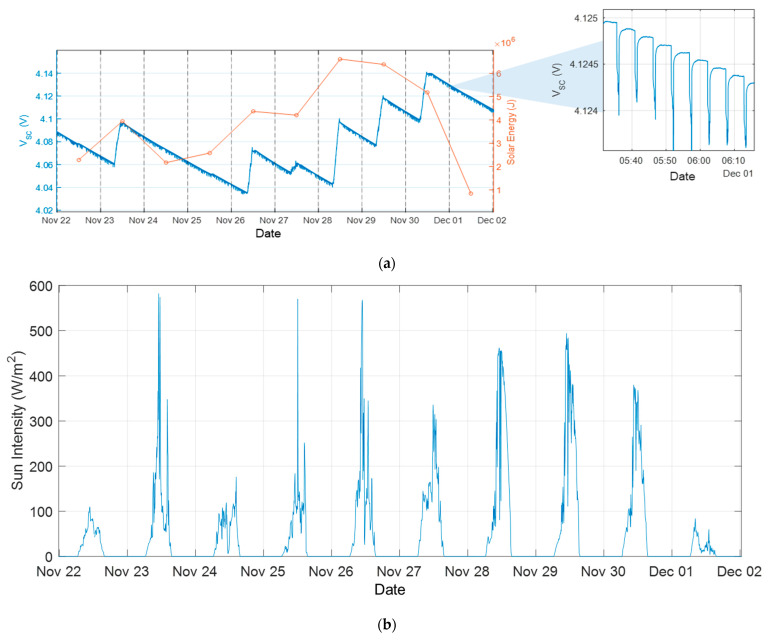

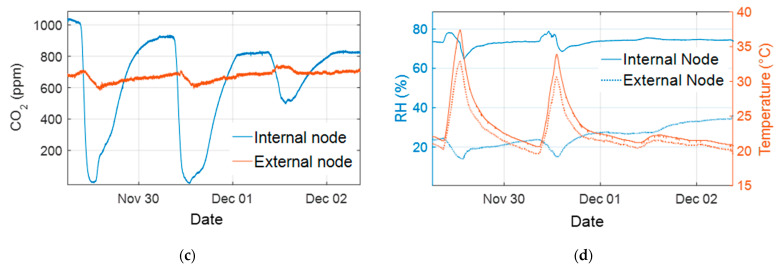
Results of the tests with plants. (**a**) V_SC_ and estimated daily solar energy from pyranometer data measured during the test. The inset shows a magnified view of V_SC_ highlighting the periodic voltage drops caused by node activation and data transmission. (**b**) Sun radiation intensity measured by the pyranometer over the same period of test. (**c**) CO_2_ concentration inside the plant chamber (blue) and in the laboratory (red); diurnal CO_2_ dynamics associated with plant photosynthesis and respiration are visible. (**d**) Temperature (red) and relative humidity (blue) measured inside the plant chamber (solid lines) and in the laboratory (dotted lines) during the same test period.

To complement the analysis of the harvesting conditions, global solar irradiance data from a nearby pyranometer station (an SMP3 thermopile pyranometer by Kipp & Zonen, Delft, The Netherlands) located near Siena, Italy, were considered. Although these measurements do not represent the exact illumination to the PV module, being taken in open-field conditions and at a distance of about 35 km, they still provide a useful qualitative indication of the daily radiation levels. In this context, the pyranometer data should be regarded as an upper estimate of the intensity potentially available to our module. Nevertheless, they allowed us to verify the consistency between the observed charging behavior of the node, the measured plant photosynthetic fluxes and the days characterized by higher or lower global irradiance, offering an additional point of reference for interpreting the energy-harvesting performance under winter conditions. During tests, the voltage at the hybrid supercapacitor was acquired using a digital multimeter (Agilent Technologies 34401A, Santa Clara, USA). Some pictures of the experimental setup are shown in Figure 8 while measurements are reported in Figure 9.

Figure 9a summarizes the energy-harvesting performance of the system over the test period by comparing the voltage across the storage hybrid supercapacitor with the solar energy estimated from pyranometer data reported in Figure 9b. Although irradiance was generally low—often below 500 W/m^2^ even on clear days and consistently below 100 W/m^2^ during unfavorable weather conditions—the correspondence between the estimated solar energy and the capacitor voltage evolution is evident. On days characterized by poor weather conditions (e.g., 24–25 November and 1 December), the global irradiance remained below 100 W/m^2^, resulting in minimal effective illumination at the window where the PV module was placed and preventing any appreciable recharge of the hybrid supercapacitor. Nonetheless, across the entire observation period, the capacitor voltage remained stable, exhibiting no net decrease and even showing a slight overall increase. This confirms that the system is capable of maintaining energy autonomy despite being operated in extremely unfavorable winter conditions. The inset highlights the periodic voltage drops associated with node activation and wireless transmission events, clearly illustrating the power consumption profile of the node and its recovery dynamics, in accordance with results shown in Figure 6.

To complement the energy analysis, Figure 9c,d report the temperature, relative humidity, and CO_2_ concentration measured both inside the plant chamber and in the laboratory. The CO_2_ dynamics inside the chamber display the expected photosynthesis–respiration cycle: CO_2_ concentrations decrease during daylight hours due to photosynthetic uptake and rise again when the available light becomes insufficient and respiration dominates. This is also in accordance with what observed in previous works [28,29,30]. The magnitude of this cycle varies depending on the incident radiation, with a markedly reduced CO_2_ drop observed on 1 December, consistent with the lower solar irradiance recorded on that day. Moreover, the experimental results show that the CO_2_ dynamics measured inside the enclosure differ from those observed by the external reference node. In particular, while the internal concentration exhibits clear diurnal trends, the external CO_2_ remains comparatively stable. This differential behavior indicates that the dominant contribution to the internal CO_2_ signal arises from plant physiological activity rather than from ambient air fluctuations.

Overall, the results demonstrate that the sensing node can reliably monitor environmental parameters and plant-driven CO_2_ dynamics while simultaneously sustaining its energy budget under highly challenging illumination and transmission conditions. These findings provide strong evidence of the feasibility and robustness of the proposed autonomous sensing system, even during the winter season when solar energy availability is at its minimum.

### 3.4. Evaluation of Self-Powered Lifetime Outdoors

**Figure 10:**
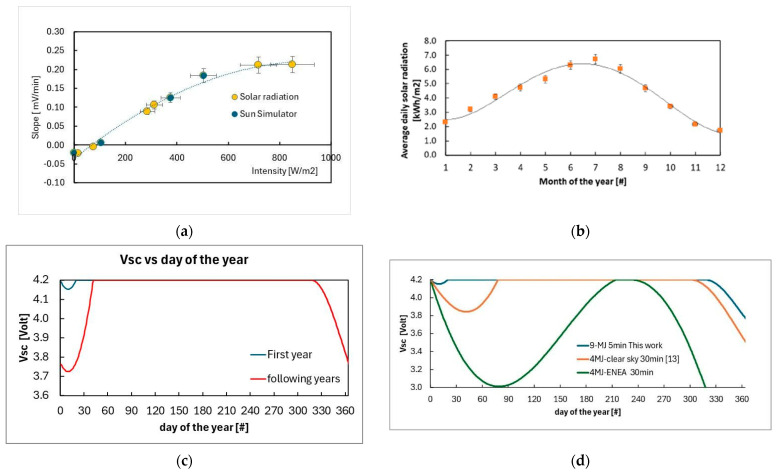
(**a**) Slope $\frac{dV_{SC}}{dt}$ vs. intensity data in case of solar and Sun Simulator exposures and best-fit curve. (**b**,**c**) V_SC_ vs. day of the year evaluated using data of plots (**a**,**b**) when operations start with full charge of the supercapacitor for transmissions every 5 min. (**d**) V_SC_ vs. day of the second and following years for transmissions every 5 min.

As discussed above, the correct operation range of the supercapacitor corresponds to V_SC_ within 3.7–4.2 V, to ensure a state of charge in the range 10–100% (see Figure 2d). The harvesting-storage system of the WSN must therefore guarantee that V_SC_ is within this range during the entire operation lifetime. We have shown in the previous sections that the change in V_SC_ during operation is related to the radiation intensity. Using data taken outdoors and under a Sun Simulator with transmissions every 5 min, we have determined a set of ($\frac{dV_{SC}}{dt}$, I) data point plotted in Figure 10a. Best fit of all data is also shown in the plot as dashed line. We observe that data taken under solar radiation and Sun Simulator generally agree. The change in sign of $\frac{dV_{SC}}{dt}$ is occurring at an intensity of about 65 W/m^2^. To perform an evaluation of the change of V_SC_ with radiation intensity on a yearly basis, we used data from ENEA Database on solar radiation in Siena (Tuscany, Italy), averaged on 2006–2022 years [31]. In particular, the average energy per unit area curve for Siena, Italy, is shown in Figure 10b. The best fit of all data shown in Figure 10a has been used to determine V_SC_ as a function of time during a typical year of insolation on this Tuscany site. Results are shown in Figure 10c,d. Starting with an initial condition in which the system is at full charge (V_SC_ = 4.2 V) on January 1st, Figure 10c shows a first decrease of V_SC_ in wintertime. Then, full charge is fully recovered in spring and maintained up to fall, where V_SC_ decreases again, reaching a minimum value of approximately 3.77 V at the end of the year. Starting from this new initial condition, the trend of the following years is shown in the same plot. It exhibits a similar seasonal evolution, with a slightly lower minimum of about 3.7 V in winter, followed by a 100% SoC achieved during springtime. The discharge profile then observed in autumn is consistent with what is observed in the first year, resulting in a V_SC_ of approximately 3.77 V at the end of the second annual cycle. Coherently, subsequent years are expected to follow the same behavior as the second year. This evaluation evidences that the system converges to a stable, periodic behavior, indicating long-term energy sustainability without cumulative discharge effects.

It is useful to compare the results obtained with the present system to those reported in [16], where a solar module composed of only four triple-junction solar cells of the same type was used. Figure 10d compares the supercapacitor voltage (V_SC_) as a function of time over one year for the current module with (i) the corresponding curve reported in [16] under clear-sky conditions, and (ii) new data calculated using the same model but based on the ENEA database for the average solar irradiance in Siena.

The comparison shows that the system proposed in [16] would not be able to operate in the correct voltage range (3.7–4.2 V) in a fully self-powered manner under realistic insolation conditions, even when using a lower transmission frequency (every 30 min) compared to the higher frequency adopted in this work (every 5 min).

The better performance of the present system is mainly due to the increased active area of the solar module, up to the total size of the PMS, and to the use of a low-power CO_2_ pulsed NDIR sensor. In fact, the system in [16] was simulating the load due to a gas sensor considering a dissipation of 10 mW during 1 min on a resistance every sensing/transmission phase. In fact, we demonstrated that our load section, comprising the low-power CO_2_ sensing device CozIR-LP, is characterized by a lower power dissipation: about 7 mW on a 20 s time frame. As a further breakthrough, in this paper the PV module input has been optimized to match the hybrid supercapacitor’s voltage. This allows for a proper charge regulation by the power management system, ensuring efficient power transfer, preventing overstressing of charging circuitry, protecting the battery from overcharging and overheating, thus rendering the system more robust in case of extended lifetimes.

## 4. Conclusions

Environmental monitoring requires sensors capable of capturing high-resolution spatiotemporal variations of key atmospheric parameters with autonomous, low-power systems. In this work we developed a wireless sensor node as a low-power platform designed for continuous environmental monitoring. The energy harvesting and storage subsystem consists of a high-efficiency PV module, a high-energy-density hybrid supercapacitor (SC) rated at 4000 F with an operating voltage range V_SC_ = 3.7–4.2 V, and a dedicated PMS. The PMS is custom-designed to accommodate the cylindrical SC (6.9 cm height, 1.2 cm radius) and occupies an area of 6.5 × 5.6 cm^2^. It is capable of delivering up to 1 A of current with an output voltage between 3.3 and 5 V. The PV module is designed to match the footprint of the PMS in order to minimize the overall system size. It consists of nine triple-junction solar cells connected in parallel, yielding an open-circuit voltage V_OC_ close to the maximum SC voltage, thereby reducing power dissipation within the PMS. The PV module has been characterized both outdoors and under a Sun Simulator. An average conversion efficiency of 27% was measured, with peak power occurring at $V_{max}$ = 4.26 V and $I_{max}$ = 98 mA under an irradiance of 600 W/m^2^.

The sensing and communication subsystem includes miniaturized CO_2_, humidity, and temperature sensors, along with a LoRa-based wireless transmission unit. The core sensing element is a compact NDIR CO_2_ sensor equipped with a solid-state infrared source and optimized for low-power operation. Laboratory calibration and validation demonstrated an effective resolution of 15–20 ppm after signal filtering, a response time of approximately 45 s (T_10−90_), and measurement errors consistent with the manufacturer’s specifications over the target CO_2_ concentration range.

The experimental validation under controlled yet realistic operating conditions confirms the robustness and feasibility of the proposed sensing system. Week-long field tests confirmed the system’s ability to operate fully autonomously, maintaining continuous functionality and reliable wireless data transmission at 5 min intervals under ambient lighting conditions. Despite extremely challenging winter illumination levels, limited direct sunlight, and a demanding communication schedule, the node maintained stable energy operation without depletion of the storage element. At the same time, it reliably monitored environmental parameters and plant-driven CO_2_ dynamics, clearly resolving the expected photosynthesis–respiration cycles and their dependence on incident solar radiation. Measurements conducted under both a solar simulator and natural sunlight, in clear and cloudy conditions, were used to determine the irradiance range for which net energy storage occurs ($\frac{dV_{sc}}{dt}\;$> 0). Energy accumulation was observed for irradiance levels I ≥ 65 W/m^2^, indicating effective operation under diffuse and low-light conditions. Furthermore, using the experimentally derived $\frac{dV_{sc}}{dt}$ versus irradiance relationship and long-term insolation data for Siena, Italy, annual simulations of V_SC_ have been performed. The V_SC_ voltage consistently remained within the correct operation range of 3.7–4.2 V and converged to a stable periodic behavior over multiple years, demonstrating long-term energy sustainability without cumulative discharge effects.

Beyond the specific application scenario considered, these results highlight the suitability of the proposed node as a general platform for energy-autonomous sensing under stringent power constraints. The effective combination of high-efficiency energy harvesting, hybrid energy storage, and low-power sensing enables continuous operation for a sensing modality that is typically challenging to support in self-powered wireless sensor networks. In particular, the adoption of a low-power LED-based NDIR CO_2_ sensor, together with an optimized energy management architecture, addresses key practical limitations associated with autonomous CO_2_ monitoring.

The selected low-cost NDIR CO_2_ sensor (CozIR) was chosen as a compromise to ensure affordability, low power consumption, and suitability for distributed deployments. As a consequence, the achievable measurement performance is inherently limited compared to laboratory-grade gas analyzers, with accuracy and resolution on the order of tens of ppm, which can be considered adequate for environmental and application-oriented monitoring. Although the sensor has been re-characterized and validated in laboratory conditions within this work, the information provided in the datasheet regarding internal signal processing, filtering, and long-term stability is limited. For this reason, additional application-dependent characterization and validation may be required prior to deployment, especially when operating conditions (e.g., temperature, humidity) significantly differ from those assumed during factory and laboratory calibration. Sensor response can indeed be influenced by environmental variables such as temperature and relative humidity. While basic compensation mechanisms are embedded in the device, residual dependencies may remain under highly variable conditions. However, in the proposed system, these effects can be eventually mitigated through additional server-side post-processing, exploiting the availability of co-located temperature and humidity measurements. Furthermore, as a self-powered node, the system performance is inherently linked to the available energy budget. The photovoltaic harvesting solution is primarily intended for outdoor or well-illuminated environments, while prolonged low-irradiance conditions may require adaptive duty-cycling strategies, trading temporal resolution for energy autonomy.

These considerations do not undermine the validity of the proposed system but rather define its intended operational domain. Future work may address extended in-field characterization and the development of server-side compensation strategies to further improve reliability under heterogeneous environmental conditions, as well as the exploration of additional application scenarios beyond plant-related monitoring.

## Figures and Tables

**Table 1:** Nominal parameters of the triple-junction solar cells for terrestrial applications [22].

Parameter	Min
Active Size [cm^2^]	1.70 × 1.64
Operating Temperature [°C]	−80 °C ÷ +80 °C
Lifetime Operation [y]	8
Weight [g]	0.7
Nominal Operating Voltage [V]	4.5
Nominal Operating Current [mA]	14.8
Efficiency [%]	29%

**Table 2:** Operational parameters of the CO_2_ CozIR-LP sensor [14].

Parameter	Min	Max
Supply Voltage [V]	−0.3	+6.0
Operating Temperature [°C]	0	+50
Pressure Range [mbar]	500	2000
Relative Humidity [%]	0	95%

## Data Availability

In the Discussion Section, this paper used solar radiation data from the Agenzia nazionale per le nuove tecnologie, l’energia e lo sviluppo economico sostenibile, dipartimento tecnologie energetiche e fonti rinnovabili C.R. ENEA Casaccia-Via Anguillarese 301, 00123 Roma (RM), Italy, http://www.solaritaly.enea.it/TabelleRad/TabelleRadIt.php (accessed on 28 August 2024).
